# External Load Variables Affect Recovery Markers up to 72 h After Semiprofessional Football Matches

**DOI:** 10.3389/fphys.2019.00689

**Published:** 2019-06-04

**Authors:** Håvard Wiig, Truls Raastad, Live S. Luteberget, Ingvar Ims, Matt Spencer

**Affiliations:** ^1^ Department of Physical Performance, Norwegian School of Sport Sciences, Oslo, Norway; ^2^ Department of Public Health, Sport and Nutrition, University of Agder, Kristiansand, Norway

**Keywords:** neuromuscular fatigue, muscle damage, performance, sprint, player monitoring

## Abstract

**Background:** Player tracking devices are commonly used to monitor external load from training and matches in team sports. Yet, how the derived external load variables relate to fatigue and recovery post-training or post-match is scarcely researched. The objective was, therefore, to investigate how external load variables affect recovery markers up to 72 h post-match.

**Methods:** Semiprofessional players from six teams wore tracking devices during three experimental football matches. External load variables including individual playing duration, total distance, PlayerLoad™, high-intensity running, and high-intensity events were derived from the tracking devices, and blood samples and performance tests from 24–59 players were undertaken post-match. The effect of the external load variables on creatine kinase, myoglobin, and countermovement jump at 1, 24, 48, and 72 h, and 30-m sprint and Yo-Yo intermittent recovery tests level 1 at 72 h post-match, were modeled. Effects were gauged as two standard deviations of the external load and interpreted as the difference between a typical high-load and a typical low-load match. The effects were evaluated with 90% confidence intervals and magnitude-based inferences.

**Results:** High-intensity running had very likely substantial effects on creatine kinase and myoglobin (moderate factor increases of 1.5–2.0 and 1.3–1.6 respectively), while duration, total distance, and HIE showed small, likely substantial effects. PlayerLoad™ and total distance had likely substantial effects on 30-m sprint time (small increases of 2.1–2.6%). Effects on countermovement jump performance were generally non-substantial. Despite these relationships, the uncertainty was too large to predict the recovery of individual players from the external load variables.

**Conclusions:** This study provides evidence that external load variables have an effect on recovery markers up to 72 h post-match. Hence, tracking external load in matches may be helpful for practitioners when managing training load and recovery strategies post-match. However, it is recommended that several different external load variables are monitored. Future research should continue to address the problem of predicting recovery from external load variables.

## Introduction

Football match load is known to cause increases in muscle damage indicators (Andersson et al., 2008), alter the biochemical milieu (Ascensão et al., 2008), and cause glycogen depletion (Bangsbo et al., 2006), leading to neuromuscular fatigue and physical performance impairment up to 72–96 h post-match (Silva et al., 2018a). In this rather long post-match period, information on the players’ recovery status could be useful in order to optimally manage training load and recovery strategies for the individual player. Measuring the recovery status directly is however time-consuming and often involves invasive measurements or performance tests that are difficult to implement in the daily training routine. Conversely, the use of player tracking technology to measure external load in training and matches is easy and requires minimal player involvement and additional assessments. The use of such technology in team sports has escalated in recent years, both in research (Malone et al., 2017) and in practical applications (Akenhead and Nassis, 2016). Global Navigation Satellite Systems (GNSS) and Local Positioning Systems (LPS) with integrated Inertial Measurements Units (IMU) provide data on position, distance, speed, and accelerative efforts as measures of external load. While shown to have good reliability (Luteberget et al., 2018a) and validity (Luteberget et al., 2018b), player tracking systems have limited value if the quantified external load is not related to performance, fatigue, or recovery.

A few studies have investigated the relationship between external load variables and recovery from football matches *via* muscle damage indicators in blood and neuromuscular fatigue measurements (Thorpe and Sunderland, 2012; de Hoyo et al., 2016; Russell et al., 2016; da Silva et al., 2018b). While these studies have reported associations between creatine kinase (CK) and high-intensity running distance, sprint distance, and number of sprints, between myoglobin (MYO) and number of sprints, and between countermovement jump performance (CMJ) and decelerations and accelerations, they are somewhat limited to correlation analyses with small sample sizes. Furthermore, from a practical point of view, there are a lack of studies investigating the specific effect of external load variables on recovery markers, both the magnitude of the effect and the recovery time back to baseline values. One exception is Rowell et al. (2017) who found a dose-response relationship of PlayerLoad™ on CMJ, but only one external load variable was investigated. Consequently, studies investigating several external load variables and also their effect on important physical performance parameters such as sprint or intermittent running performance are needed.

Seventy-two hours post-match is a key time-point where the next match or a hard training session may take place. Most studies have examined the relationships for only 24–48 h post-match (Thorpe and Sunderland, 2012; de Hoyo et al., 2016; Russell et al., 2016; da Silva et al., 2018b), despite evidence showing substantial changes in recovery markers at 72 h post-match (Ascensão et al., 2008; Ispirlidis et al., 2008). Additionally, due to individual differences in recovery time, some players might be recovered and some players not, hence being able to predict the recovery status on day three post-match is practically important.

The objective of the current study was therefore to investigate how external load affects recovery up to 72 h after a football match. External load was quantified as playing duration, high-intensity events (HIEs), high-intensity running distance (HIR), PlayerLoad™, and total distance covered. Recovery was operationalized into recovery markers for muscle damage indicators (CK and myoglobin, MYO); neuromuscular function (countermovement jump, CMJ); sprint performance (30-m sprint, SP30); and intermittent endurance performance (Yo-Yo Intermittent Recovery test level 1, YOYO). A secondary objective was to investigate how different amounts of external load affect the recovery status 72 h post-match.

## Materials and Methods

### Participants

**Figure 1:**
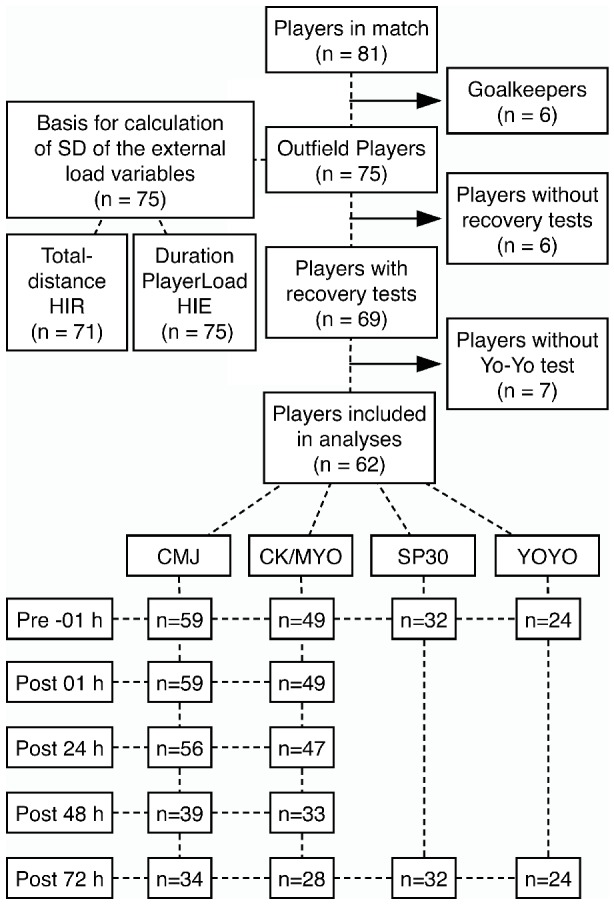
Flow chart showing number of participants included in (1) the analyses of the different recovery markers on each time-point and (2) the calculation of SD which were used for the rescaling of the external load variables.

Seventy-five outfield male football players from six Norwegian second division teams participated in the study, of whom subject characteristics are summarized in Table 1. The players reported an average of 7.6 ± 2.3 training sessions per week (matches excluded) for a typically in-season week, with 80% of the players reporting “less” or “somewhat less” training load in the last week before their experimental match. The number of players included in the different analyses is highlighted in Figure 1.

**Table 1:** Summary of subject characteristics and baseline values for recovery markers.

Characteristic	*n*	Mean	SD	Min	Max
**Subject characteristics**					
Age (yr)	75	20.4	4.6	16	45
Height (cm)	75	178.0	6.1	164	194
Body mass (kg)	75	72.7	7.2	49	96
**Baseline values**					
CK (U/L)	49	367	273	59	1,600
MYO (μg/L)	49	39	37	21	256
CMJ (cm)	59	43.0	4.5	33.2	57.5
SP30 (s)	32	4.27	0.18	3.62	4.53
YOYO (m)	24	2,000	388	1,200	2,800

### Study Design

The study took place 14–23 days after their last match of the season. It consisted of three experimental matches (one per team) with corresponding familiarization-, pre- and post-match tests conducted at −144, −72, −1, 1, 24, 48, and 72 h relative to the matches. When conducted on the same day, the test order was: blood samples, CMJ, SP30, and YOYO. The players were instructed to refrain from other intense physical exercises within the study period and to follow their normal preparation before the match regarding nutrition and sleeping strategies. The matches were preceded by a standardized 40-min warm-up consisting of 5 min of jogging, the CMJ test procedure, team-organized running drills, and a play exercise. Standard 90-min matches were officiated according to FIFA rules, and teams and players were instructed to give their best to win. Immediately after the match, the players consumed a 330-ml recovery drink (Yt Restitusjonsdrikk, TINE, Norway). In accordance with the study objectives and typical substitution practices in official matches, two to three pre-planned substitutions at 45 and 60 min were implemented per team to spread the match load from low to high values.

### Recovery Testing Procedures

Venous blood samples were drawn, centrifuged at 4°C for 10 min at 1300 g, and stored in −80° until analyzed for CK and MYO at the Oslo University Hospital, Rikshospitalet (Oslo, Norway; Cobas 8,000, Roche Diagnostics, USA). The laboratory’s stated coefficient of variation (CV) is 5 and 6% for CK and MYO respectively. Baseline values were taken from the −1-h blood sample.

CMJ, with hands placed on hips, was performed on a portable force platform (FP4, HUR labs, Tampere, Finland) and jump height was analyzed by the provided software (Force Platform Software Suite, Version 2.6.51, Kokkola, Finland). Data from our lab show a CV of 4.7%. The warm-up procedure consisted of a 5-min jog followed by three jumps with 80, 90, and 100% effort. Each player performed three to five jumps, interspersed with 15 s of rest, where the highest jump was used for analyses. The best of the −72- and −1-h CMJ was used as the baseline value.

SP30 was conducted with error correction processing timing gates (SmartSpeed Pro, Fusion Sport, Brisbane, Australia) placed at 0 and 30 m, and with a starting position 0.3 m before the first gate. Participants were instructed to start in a static, forward leaning position, and then sprint as fast as possible past a cone placed at 35 m. The best of three trials, with minimum 2-min rest between, was exported for analysis. Reliability testing from our lab shows a CV of 1.7%. Baseline values were taken from the −72-h SP30 test.

The YOYO test was conducted according to the instructions described by Krustrup et al. (Krustrup et al., 2003). A specific warm-up consisting of the 11 first stages of the test were undertaken, followed by a 2-min rest. The total distance in meters was used in the analysis. Furthermore, the best of the pre- and post-results (YOYOmax) was used as a measure of the players’ aerobic fitness. The test-retest CV is shown to be 4.9% (Krustrup et al., 2003). Baseline values were taken from the −72-h YOYO test.

### Tracking of External Load

All three matches were played in the same indoor football stadium (105 m by 65 m) with a third-generation artificial turf, temperature of 15 ± 1°C, and a relative humidity of 77 ± 5%. The players wore two different tracking devices, one IMU device (OptimeEye S5, Catapult Sports, Australia, with GNSS turned off) and one LPS device (ClearSky T5, Catapult Sports, Australia). These devices were taped together, with the IMU closest to the body and located between the scapulae in a manufacturer-provided vest (Catapult Sports, Australia). All IMU devices were calibrated according to the manufacturer’s instructions. The LPS was set up with 18 anchor nodes fixed around the pitch, and spatial calibration was carried out according to manufacturer’s recommendations. Three players missed LPS data due to signal problems and one due to limited available LPS devices.

### Data Processing

Five different external load variables were chosen to provide different representations of the actual match load. Playing duration (on field time), PlayerLoad™, and HIE were extracted from the IMU device using the Sprint software (version 5.1.7, Catapult Sports, Australia), and total distance and HIR were extracted from the LPS using the Openfield Software (version 1.12, Catapult Sports, Australia). PlayerLoad™ is a vector magnitude expressed in arbitrary units as the square root of the sum of the squared instantaneous rate of change in acceleration in three dimensions, described more comprehensively by Boyd et al. (2011). HIE is the sum of acceleration, deceleration, and change of direction events exceeding a threshold of 2.5 m/s based on procedures by Luteberget and Spencer (Luteberget and Spencer, 2017). During indoor field assessment, HIE and PlayerLoad™ have shown an inter-device CV of 3.1 and 0.9% respectively (Luteberget et al., 2018a). HIR is the total distance covered with running speed over 19.8 km/h, while total distance is the total distance covered at any speed. A validity study using the same LPS system as the current study has shown a 2–4% error in linear and nonlinear distance when conducted in an indoor environment (Sathyan et al., 2011).

### Statistical Analysis

The recovery markers were log-transformed, to reduce bias due to nonuniformity of errors, before being analyzed as change-scores using a linear mixed model (MIXED procedure in SAS 9.4 Software; SAS Institute, Cary, NC, USA). The effects were back-transformed to express factor or percent changes. Time, Time × external load variable, Time × baseline, and Time × YOYOmax were specified as fixed effects, with Time treated as nominal variable. When YOYO was the dependent variable, YOYOmax was omitted from the model because it contained partly the same numbers as YOYO baseline. To deal with interdependency and unequal variances in the models with repeated measurements (CK, MYO, and CMJ), the R matrix were specified with Time, PlayerID as blocks and an “unstructured” covariance structure, using the REPEATED statement in SAS. SP30 and YOYO had no repeated measurements and were analyzed without a REPEATED statement. Separate analyses were done for each external load variable for every recovery marker. The main effect of interest, Time × match load, was adjusted for baseline to address the regression to the mean effect, and YOYOmax to address the possibility of fitness being a confounder affecting both match load (Krustrup et al., 2003; Bradley et al., 2013; Redkva et al., 2018) and recovery (Johnston et al., 2015). Furthermore, to properly evaluate the magnitude of the effect of continuous variables, they were rescaled by dividing by two standard deviations (SDs). Two SDs also correspond approximately to the mean separation of lower and upper tertiles (Hopkins et al., 2009), and can be justified as a separation of typically high and low match loads. The magnitude of the effects is presented as standardized effect sizes (ES: the effects divided by the SD of the baseline value), where <0.2, 0.2–0.6, 0.6–1.2, 1.2–2.0, and >2.0 are regarded as trivial, small, moderate, large, and very large effects respectively. Nonclinical, magnitude-based inferences were used, where an effect was deemed unclear if the 90% confidence interval included small positive and negative effects; the effect was otherwise deemed clear. Qualitative assessment of chances of clear outcomes were as follows: >25–75%, possibly; >75–95%, likely; >95–99%, very likely; >99%, most likely (Hopkins et al., 2009).

## Results

### Match Load

As a result of substitutions, the match load across all players was spread in a linear manner for all external load variables, except for duration where 61% of the players played a full 90-min match. Descriptive summaries of total and relative match load are shown in Table 2.

**Table 2:** Summary of total match load and match load per minute for selected external load variables, for all players and for different groups of players (mean ± SD).

Group	*n*	Duration (min)	Distance (m)	PlayerLoad™ (AU)	HIE (#)	HIR (m)
**Total match load**						
All	75	72.7 ± 24.9	8,305 ± 2,627	780 ± 290	152 ± 62	380 ± 200
Entire match	44	91.2 ± 1.0	10,110 ± 972	966 ± 174	185 ± 52	434 ± 199
Replaced	16	54.7 ± 16.8	6,673 ± 2,016	637 ± 191	124 ± 43	357 ± 205
Substitute	15	37.4 ± 13.7	4,483 ± 1,075	386 ± 123	85 ± 31	237 ± 113
**Match load per min**						
All	75		116 ± 14	10.8 ± 1.8	2.2 ± 0.6	5.4 ± 2.6
Attackers	10		112 ± 7	10.0 ± 1.2	2.1 ± 0.4	5.0 ± 2.3
Central defenders	14		101 ± 5	9.1 ± 1.0	1.7 ± 0.5	3.2 ± 1.3
Central midfielders	22		128 ± 12	12.2 ± 1.9	2.4 ± 0.5	5.0 ± 2.3
Fullbacks	13		112 ± 14	10.6 ± 1.0	2.0 ± 0.5	6.0 ± 1.6
Wide midfielders	16		117 ± 12	11.3 ± 1.5	2.4 ± 0.5	8.0 ± 2.7

### Mean Change in Recovery Markers

**Figure 2:**
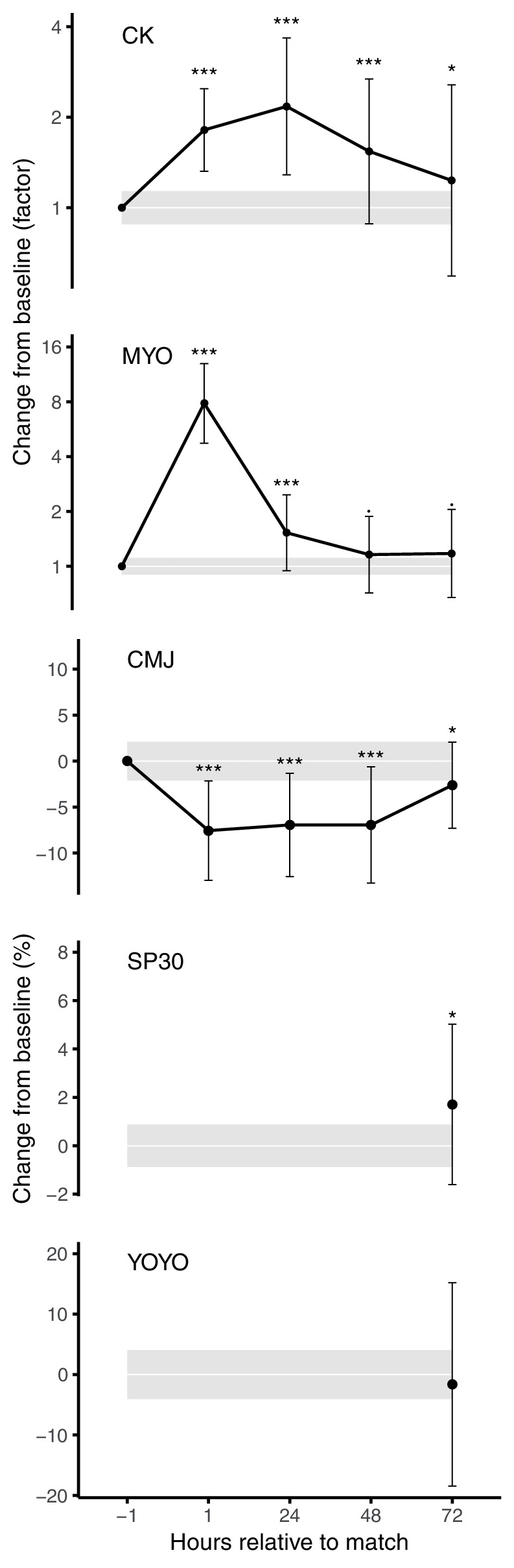
Factor or percent change from baseline for the specific recovery markers in the hours post-match, adjusted for baseline, PlayerLoad^TM^, and YOYOmax. The spread is indicated by SD and shaded area represents trivial changes. Probabilistic statements about the true effect are labeled as follows: · = possibly, * = likely, and *** = most likely.

Baseline values of the recovery markers are shown in Table 1, and the mean changes in recovery markers from pre- to 1, 24, 48, and 72 h post-match are presented in Figure 2. The matches induced most likely substantial increases in CK at 1 h (ES = 0.92), 24 h (ES = 1.20), and 48 h (ES = 0.67) post-match, whereas a likely substantial increase was seen 72 h post-match (ES = 0.32). Myoglobin peaked at 1 h post-match with a most likely substantial increase (ES = 3.80), followed by a most likely substantial increase at 24 h (ES = 0.78), and possibly substantial increases at 48 h (ES = 0.27) and 72 h (ES = 0.30). CMJ height showed a most likely substantial decrease at 1, 24, and 48 h and a likely substantial decrease at 72 h post-match with ES of −0.75, −0.68, −0.68, and −0.25 respectively. SP30 showed a likely substantial increase (ES = 0.38) at 72 h post-match, while for YOYO, the effect was trivial and unlikely substantially positive (ES = −0.08).

### The Effects of External Load Variables on Recovery Markers

**Figure 3:**
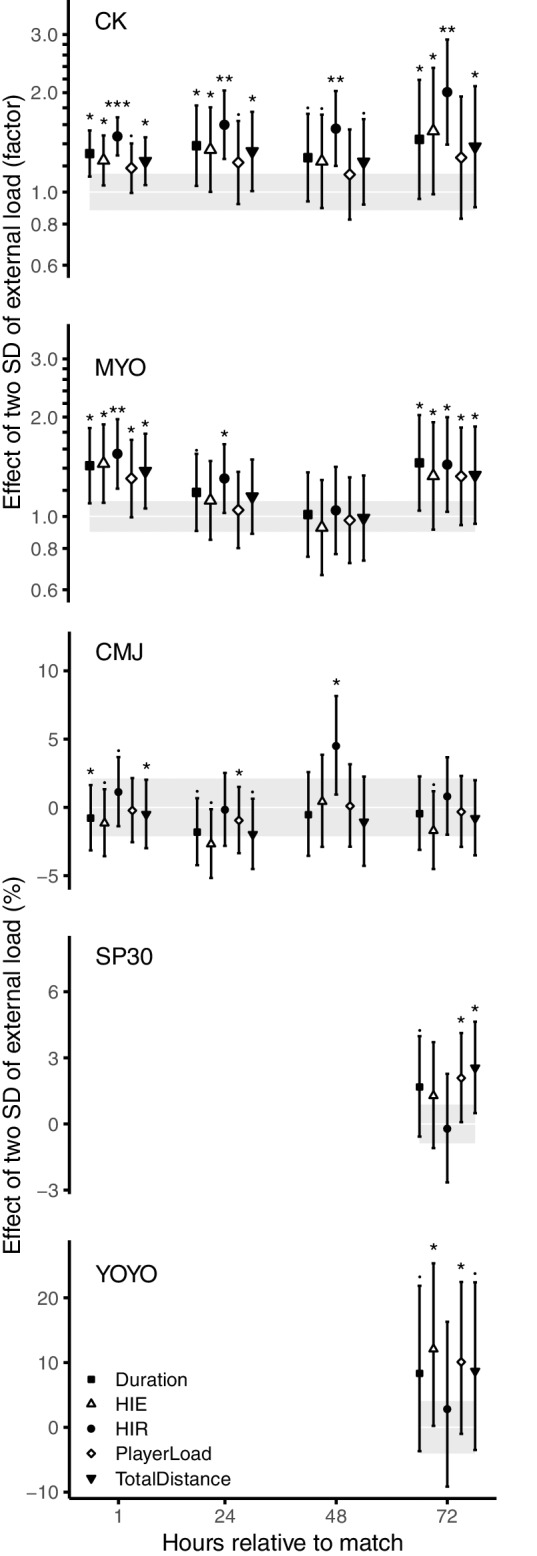
The factor or percent effect of two SDs of match load on recovery markers at the specific time-points, adjusted for baseline and YOYOmax. Two SDs of match load are interpreted as the difference between matches with typical high and low load. Uncertainty in the estimates is indicated by 90% confidence intervals and shaded area represents trivial changes. Probabilistic statements about the true effect are labeled as follows: · = possibly, * = likely, ** = very likely, and *** = most likely.

The effects of the external load variables on recovery markers at each time-point are presented in Figure 3. The external load variables had positive effects on the muscle damage indicators. HIR had the strongest relationship with CK showing very likely to most likely substantial effects, consistent throughout all time-points (ES = 0.60–1.08). Duration, total distance, and HIE showed likely substantial effects on CK at 1 h (ES = 0.33–0.42), 24 h (ES = 0.44–0.50), and 72 h (ES = 0.49–0.66). The effects on MYO at 1 h post-match was very likely substantial for HIR (ES = 0.80) and likely substantial for duration (ES = 0.65), HIE (ES = 0.68), total distance (ES = 0.58), and PlayerLoad™ (ES = 0.49). Except for a likely substantial increase of HIR (ES = 0.49) and a possibly substantial effect of Duration (ES = 0.31) at 24 h, the other effects at 24 h and 48 h post-match were unclear. At 72 h, likely substantial effects on MYO were found for all variables (ES = 0.52–0.69). The observed effects on CMJ were generally trivial or unclear, except for a possibly substantial negative effect of HIE at 24 h (ES = −0.26) and a likely substantially positive effect of HIR at 48 h post-match (ES = 0.40). SP30 performance 72 h post-match was affected negatively by total distance (ES = 0.56) and PlayerLoad™ (ES = 0.46), showing likely substantially negative effects. On the contrary, likely substantially positive effects of HIE (ES = 0.56) and PlayerLoad™ (0.47) were seen on YOYO performance 72 h post-match.

### Effect of External Load Variables on Recovery Status 72 h Post-match

**Figure 4:**
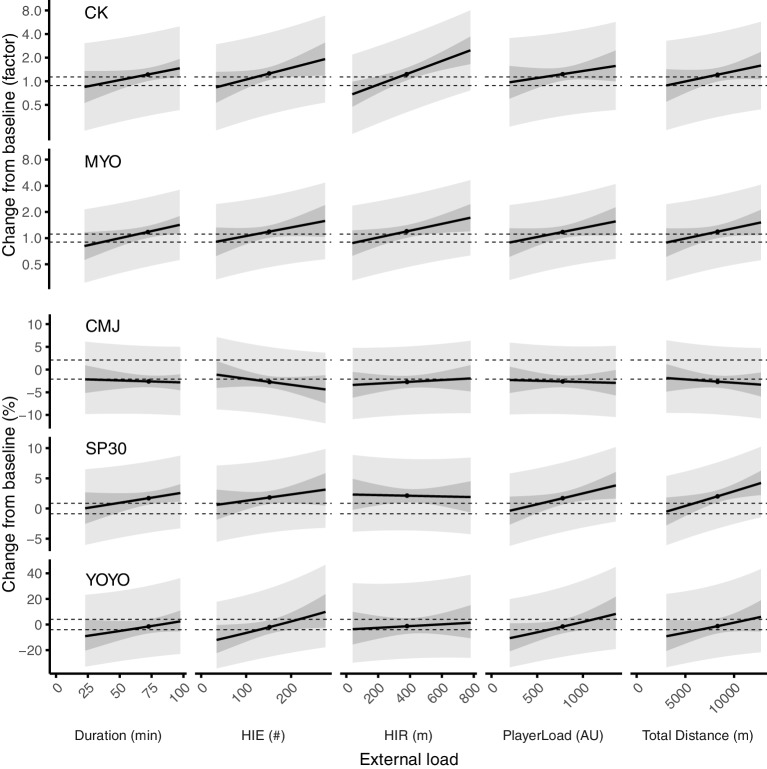
Predicted mean change in recovery markers at 72 h post-match for different amounts of match load, after adjustments for baseline and YOYOmax. Match load values are within the range of match load values in this study and mean match load is indicated by a dot symbol. The area between the dotted lines indicates trivial changes; the dark shaded area shows 90% confidence interval, and the light shaded area shows 90% prediction interval.

The predicted mean changes in recovery markers at 72 h for given match loads are depicted in Figure 4. External load variables that are substantially affecting recovery markers are highlighted in Figure 3. Other external load variables were non-substantial meaning that a change in match load could cause either trivial change, or substantial increase or decrease in the recovery markers. While substantial effects were seen on predicted means for some external load variables, prediction intervals for individual values covered both substantially negative and substantially positive values throughout the range of match load on all external load variables.

## Discussion

This study investigated how external load variables, derived from player tracking devices, affected subsequent recovery up to 72 h post-match. The external load variables were found to impact both the magnitude and the length of the recovery. HIR was the strongest predictor of muscle damage indicators, while PlayerLoad™ and total distance predicted recovery of sprint performance, and HIE and PlayerLoad™ predicted YOYO performance. Unexpectedly, recovery of CMJ performance could not be predicted. Despite these substantial mean effects, external load variables were not able to predict recovery in individual players.

### Impact on Muscle Damage Indicators

The increases observed in CK and MYO post-match indicates muscle damage which could be categorized as mild exercise-induced muscle damage (Paulsen et al., 2012). The response is comparable to other studies with reserve teams (Thorpe and Sunderland, 2012; Russell et al., 2016) and professional players (Silva et al., 2013), despite that the mean match duration, total- and high-intensity distance were lower than observed in a typical full match (Bradley et al., 2013). Furthermore, the response was higher than reported by de Hoyo et al. (2016), who also included substitutes in their analysis. These comparisons suggest a high response of muscle damage indicators in the current study. Following the same patterns as in a recent meta-analysis (Silva et al., 2018a), CK and MYO peaked at 24 and 1 h post-match, respectively, and an increase from baseline was still evident after 72 h for CK. Large variations were observed at 72 h, meaning that the muscle damage indicators had returned to baseline in some players, but not in others. For example, two players still had increasing CK at 72 h to over 3,200 U*L^−1^, suggesting a more severe muscle damage (Paulsen et al., 2012).

This is the first study modeling the effect of different external load variables on recovery markers, for a full 72-h time period post-match in football players. The effects, understood as the difference between a typical high and low match load, provide evidence that match load explains changes in the two indicators of muscle damage (Figure 3). Of the five external load variables, HIR was the strongest predictor, consistent throughout all time-points. The larger effect of HIR is supported by other studies where change in CK correlated with high-intensity distance and number of sprints, but not for total distance (Thorpe and Sunderland, 2012; de Hoyo et al., 2016; Russell et al., 2016). The reason for the larger effect could be the high-force and high-speed muscle contractions occurring when maintaining or decelerating from high running speeds, causing muscles to work while lengthening. Such eccentric muscle contractions are shown to cause tearing and disruption of muscle fibers (Paulsen et al., 2012). HIE and PlayerLoad™, that are based on accelerometer data, could hypothetically assess football-specific movements such as accelerations, decelerations, and change of directions to a higher degree than for example distance covered. Instead, our data show that HIE had a lower effect than HIR on CK and MYO, suggesting that running speed is an important factor for muscle damage. PlayerLoad™ on the other hand had the lowest effects which makes it a poor predictor of muscle damage indicators.

### Impact on Neuromuscular Fatigue

The observed decrease in CMJ performance suggests a neuromuscular fatigue comparable to other studies (Nedelec et al., 2014). Unexpectedly, the decrease in CMJ could not be explained by any of the external load variables in contrast to Rowell et al. (2017) where a dose-response relationship was found between low, medium, and high PlayerLoad™ groups and CMJ height 0.5 and 18 h post-match. Moreover, Russell et al. (2016) found moderate correlations between change in peak power output from CMJ and high-intensity running distance and sprint distance.

Other studies have found short-lived relationships between change in CMJ and high-intensity accelerations (Russell et al., 2016), hard changes of directions (Nedelec et al., 2014) at 24 h, and decelerations at 0.5 h and at 48 h (de Hoyo et al., 2016). These relationships suggest that CMJ performance could be linked to accelerative efforts that target the same muscles that are active in CMJ. Although we did find a possibly small effect of HIE on CMJ at 24 h, the uncertainty in the estimates and inconsistency over the time-points does not provide strong evidence for such relationship. Hence, one might also question if these specific variables really are able to identify the true match load that causes neuromuscular fatigue.

### Impact on Sprint and Intermittent Endurance Performance

The decreased SP30 performance at 72 h post-match indicates that sprint performance is not recovered 3 days post-match, in line with some studies (Ispirlidis et al., 2008; Fatouros et al., 2010), but not all (Silva et al., 2013). PlayerLoad™ and total distance showed small effects on SP30 at 72 h. To our knowledge, no other studies have examined such relationship. As opposed to muscle damage, which was affected by high-intensity work, SP30 was affected by variables describing match load volume. In line with this finding, it has been proposed that recovery of sprint performance could be linked to the duration of exercise, as basketball and handball have shown shorter recovery times than football (Doeven et al., 2018). For YOYO, no substantial change was found from baseline to 72 h post-match. Nevertheless, positive effects of HIE and PlayerLoad™ were still found, suggesting that higher match load improves the YOYO performance 72 h. The reason could be that a conditioning effect, due to that the match was played a couple of weeks after the season, was evident for the players with the highest match load, while not in the players with the lowest match load. Such conditioning effect could be explained by fitter players perform more running activity (Krustrup et al., 2003), but also recover faster (Johnston et al., 2015).

### Match Load as a Predictor of Recovery Status

The substantial effects of external load variables on CK, MYO, and SP30 that were seen at 72 h post-match provide evidence that match load affects the time to recovery. Thus, players with low match load could recover at 72 h, while players with high match load could not. Such a finding has important practical applications for teams using tracking devices when managing recovery strategies or training load. Moreover, our data showed that some external load variables could predict recovery on average, but not in individuals based on the wide prediction interval (Figure 4). The wide prediction intervals seen in Figure 4 are a consequence of large individual differences in the recovery, as indicated by the SD in Figure 2. Some of the variability in the recovery might be explained by differences in the individual player’s relative match load, i.e., the current match load compared to his typical match load over several matches. Given the large within-player, match-to-match variation in external load seen in football (Carling et al., 2016; Al Haddad et al., 2018), some players had presumably a higher relative external load, while others had lower relative load. A multiple-match design must be carried out to address if differences in within-player external load could predict the recovery from match more reliably than between-player external load.

## Limitations

A limitation of the study is the aforementioned one match per player design, which only allowed for between-player modeling of the external load variables. A multiple-match design that models the effect of within-player match load could possibly have decreased the uncertainty in the estimates of recovery. In addition, we suspect that the study, especially on the last two time-points, was somewhat underpowered as some of the measures were inherently unreliable. Although the external load and recovery data were regarded as representative, the matches were nonofficial matches, played 2–3 weeks after the season, in a period without other matches and with a lower self-reported training load. Hence, the match load and the recovery from the match might have been different from an official, within-season match. Lastly, the control of the players’ physical activity, nutrition strategies, and sleep before and after the match were limited to pre-study instructions from the research staff.

## Conclusions and Practical Applications

This study provides evidence that external load variables derived from player tracking systems have an effect on recovery markers up to 72 h post-matches. Such information may help practitioners when managing training load and recovery strategies post-match. HIR had the most substantial effect on muscle damage indicators, and PlayerLoad™ and total distance affected sprint performance. Hence, a combination of several different tracking device variables is advised to ensure a better representation of the match load. An unexpected finding, which requires further investigation, was the trivial effect of external load variables on CMJ. While the mean changes in recovery markers approached baseline values at 72 h post-match, the effects of external load variables on the same recovery markers were still substantial, suggesting that external load variables could partly explain the time to recovery. Despite these substantial effects, it was not possible to predict the recovery of individual players at 72 h from any of the external load variables due to too much uncertainty in the predictions.

## Ethics Statement

This study was carried out in accordance with the recommendations of Regional Committees for Medicine and Health Research Ethics. All subjects gave written informed consent in accordance with the Declaration of Helsinki. The study’s data storage methods were approved by the Norwegian Centre for Research Data.

## Author Contributions

HW, TR, and MS were responsible for the idea and the design of the study. Data were collected by HW, LL, II, and MS. HW was responsible for the data analyses and drafted the manuscript. All authors contributed to the interpretation of the results, critical revision, and approval of the final version.

### Conflict of Interest Statement

The authors declare that the research was conducted in the absence of any commercial or financial relationships that could be construed as a potential conflict of interest.
